# Carnosine Prevents Aβ-Induced Oxidative Stress and Inflammation in Microglial Cells: A Key Role of TGF-β1

**DOI:** 10.3390/cells8010064

**Published:** 2019-01-17

**Authors:** Giuseppe Caruso, Claudia G. Fresta, Nicolò Musso, Mariaconcetta Giambirtone, Margherita Grasso, Simona F. Spampinato, Sara Merlo, Filippo Drago, Giuseppe Lazzarino, Maria A. Sortino, Susan M. Lunte, Filippo Caraci

**Affiliations:** 1 Oasi Research Institute—IRCCS, 94018 Troina, Italy; mcgiambirtone@oasi.en.it (M.Gi.); grassomargherita940@gmail.com (M.Gr.); 2 Ralph N. Adams Institute for Bioanalytical Chemistry, University of Kansas, Lawrence, KS 66047-1620, USA; forclaudiafresta@gmail.com (C.G.F.); slunte@ku.edu (S.M.L.); 3 Department of Pharmaceutical Chemistry, University of Kansas, Lawrence, KS 66047-1620, USA; 4 Bio-nanotech Research and Innovation Tower (BRIT), University of Catania, 95125 Catania, Italy; nmusso@unict.it; 5 Department of Drug Sciences, University of Catania, 95125 Catania, Italy; 6 Department of Biomedical and Biotechnological Sciences, Section of Pharmacology, University of Catania, 95125 Catania, Italy; simona_spampinato@hotmail.com (S.F.S.); sara_merlo@hotmail.com (S.M.); f.drago@unict.it (F.D.); msortino@unict.it (M.A.S.); 7 Department of Biomedical and Biotechnological Sciences, Division of Medical Biochemistry, University of Catania, 95125 Catania, Italy; lazzarig@unict.it; 8 Department of Chemistry, University of Kansas, Lawrence, KS 66047-1620, USA

**Keywords:** carnosine, microglia, Alzheimer’s disease, neurodegeneration, neuroinflammation, reactive oxygen and nitrogen species, oxidative stress, TGF-β1

## Abstract

Carnosine (β-alanyl-L-histidine), a dipeptide, is an endogenous antioxidant widely distributed in excitable tissues like muscles and the brain. Carnosine is involved in cellular defense mechanisms against oxidative stress, including the inhibition of amyloid-beta (Aβ) aggregation and the scavenging of reactive species. Microglia play a central role in the pathogenesis of Alzheimer’s disease, promoting neuroinflammation through the secretion of inflammatory mediators and free radicals. However, the effects of carnosine on microglial cells and neuroinflammation are not well understood. In the present work, carnosine was tested for its ability to protect BV-2 microglial cells against oligomeric Aβ1-42-induced oxidative stress and inflammation. Carnosine prevented cell death in BV-2 cells challenged with Aβ oligomers through multiple mechanisms. Specifically, carnosine lowered the oxidative stress by decreasing NO and O_2_^−•^ intracellular levels as well as the expression of iNOS and Nox enzymes. Carnosine also decreased the secretion of pro-inflammatory cytokines such as IL-1β, simultaneously rescuing IL-10 levels and increasing the expression and the release of TGF-β1. Carnosine also prevented Aβ-induced neurodegeneration in mixed neuronal cultures challenged with Aβ oligomers, and these neuroprotective effects were completely abolished by SB431542, a selective inhibitor of the type-1 TGF-β receptor. Our data suggest a multimodal mechanism of action of carnosine underlying its protective effects on microglial cells against Aβ toxicity with a key role of TGF-β1 in mediating these protective effects.

## 1. Introduction

Carnosine (β-alanyl-L-histidine) is a natural dipeptide widely distributed in mammalian tissues [1,2] and exists at particularly high concentrations (millimolar order) in the brain as well as in skeletal and cardiac muscles (up to 20 mM). Carnosine has been shown to be neuroprotective through different mechanisms: the prevention of oxidative stress [3], reduction of intraneuronal amyloid-βeta (Aβ) accumulation, mitochondrial dysfunctions and cognitive deficits in 3xTg-AD mice [4], as well as the inhibition of Aβ aggregation [5], modulation of macrophage nitric oxide (NO) production and pro-/anti-inflammatory (M1/M2) ratio [6]. Furthermore, it is also able to scavenge the superoxide ion (O_2_^−•^) [7] and other reactive species [8].

Aβ is a 42 amino acids long (Aβ1-42) peptide physiologically present in the brain and cerebrospinal fluid of human beings [9]. Together with marked inflammation [10], both the extracellular deposition of insoluble aggregates of this peptide in the brain and its blood vessels [11,12] and the formation of neurofibrillary tangles composed by the highly phosphorylated form of tau protein [13] represent the neuropathological hallmarks of Alzheimer’s disease (AD). Aβ peptide can undergo aggregation through a step-by-step process, starting with soluble monomers and evolving to the formation of oligomers, protofibrils, and mature fibrils [14], with the oligomeric structures representing the more toxic species [15].

Microglia are a subtype of brain glial cells and constitute up to 10% of all cells in the healthy human cortex [16]. These cells are in intimate contact with neurons and are involved in many basic physiological processes [17]. Microglial cells, often found near Aβ plaques in AD patients [18], are able to produce various neurotrophic and anti-inflammatory factors essential for cell growth and protection; they can also release different cytotoxic substances, such as reactive oxygen species (ROS) and reactive nitrogen species (RNS), and pro-inflammatory cytokines such as IL-1β, IL-6, and TNF-α [17]. All the above substances are strongly connected to AD pathogenesis and amyloid-related neurodegeneration [19].

NO (RNS species) and O_2_^−•^ (ROS species) are part of the natural aerobic metabolism of cells and are involved in many physiological and pathological processes [20,21]. Peroxynitrite, the reaction product between NO and O_2_^−•^, can react and consequently damage fatty acids, proteins, DNA, and mitochondria leading to oxidative and nitrosative stress [22], inflammation [23], and then neurodegenerative phenomena [24,25]. During the inflammation process, both inducible nitric oxide synthase (iNOS), responsible for NO production [26,27], and NADPH oxidase (Nox), responsible for O_2_^−•^ production [28], are overactivated in immune cells such as macrophage and microglia [20,29,30]. When simultaneously activated, iNOS and Nox act synergistically to promote neuronal death through the generation of peroxynitrite, which is a more dangerous species if compared with NO and/or O_2_^−•^ [31]. Mediators of cytotoxicity released from activated microglia also include arachidonic acid, glutamate, and histamine [32]. Furthermore, it has been shown that macrophages and microglia play a crucial role in several diseases characterized by oxidative stress and inflammation and also that the modulation of their pro-/anti-inflammatory (M1/M2) ratio and secretion products might represent a novel pharmacological approach for the treatment of these disorders [33,34].

Neuroinflammation is a widely accepted factor associated with the pathogenesis of AD [35]. Different cell types, including microglia, shift from the resting to activated state during the neuroinflammation process [36], producing a higher amount of pro-inflammatory cytokines such as TNF-α, IL-1β, and IL-6 [19]. Activated microglia have been shown to contribute to the development and progression of neurodegenerative disorders [37] and the presence of both reactive microglia and astrocytes has been observed in association with amyloid accumulation in AD brain [38]. However, activated microglia are also able to produce anti-inflammatory mediators [39]. Among these molecules, the multifunctional cytokine TGF-β1 has been shown to play a pivotal role in AD, exerting neuroprotective effects against Aβ-induced neurodegeneration [40,41]. Furthermore, the secretion of TGF-β1 from peripheral blood mononuclear cells in the circulation [42] along with the levels of TGF-β1 in the plasma [43] are reduced in AD subjects. Lastly, Wyss-Coray et al. showed that TGF-β1 promoted microglial Aβ clearance and the reduction of plaque burden in AD mice and enhanced Aβ clearance by BV-2 microglial cells [44], suggesting a link between microglia activity, TGF-β1 release, and the neuroprotective activity of this neurotrophic factor against Aβ-induced toxicity.

In the present study, we first investigated the toxicity and the production of NO and O_2_^−•^ induced by different concentrations of Aβ1-42 oligomers, in the absence or in the presence of carnosine, in BV-2 cells, an established experimental model for mimicking neuroinflammation in primary microglia [45]. Additionally, in order to understand the molecular mechanisms underlying the ability of carnosine in decreasing the production of molecules related to oxidative and nitrosative stresses, we studied the expression of iNOS and Nox enzymes along with the expression and secretion of pro- and anti-inflammatory cytokines in BV-2 cells challenged with Aβ1-42 oligomers. Lastly, the protective activity of carnosine, as well as the role played by TGF-β1 in preventing Aβ-induced neuronal death, was evaluated in mixed neuronal cultures.

The evidence that carnosine exerts protective effects by decreasing Aβ1-42-induced toxicity in microglial cells, counteracting the oxidative stress and the inflammation status, is presented.

## 2. Material and Methods

### 2.1. Materials and Reagents

Microglial BV-2 cells (ICLC ATL03001) were purchased from Interlab Cell Line Collection (ICLC, Genova, Italy). HFIP-treated amyloid β-peptide (1-42) was obtained from Bachem Distribution Services GmbH (Weil am Rhein, Germany). DMEM/F12 (1:1) medium, RPMI-1640 medium, phenol red-free RPMI-1640 medium, trypsin-EDTA solution (0.25% Trypsin/0.53 mM EDTA in HBSS without calcium or magnesium), fetal bovine serum (FBS), and penicillin–streptomycin antibiotic solution were purchased from American Type Culture Collection (ATCC) (Manassas, VA, USA). L-Carnosine, anhydrous dimethyl sulfoxide (DMSO), trypan blue solution, glucose, cytosine-D-arabinoside, sodium dodecyl sulfate (SDS), MTT [3-(4,5-dimethylthiazol-2-yl)-2,5-diphenyltetrazolium bromide] tetrazolium salt, and phosphate-buffered saline (PBS) were all supplied by Sigma Aldrich (St. Louis, MO, USA). Agilent DNA 1000 Kit was obtained from Agilent (Santa Clara, CA, USA). The 4-amino-5-methylamino-2’,7’-difluorofluorescein diacetate (DAF-FM DA) and MitoSOX Red probes were purchased from Life Technologies (Carlsbad, CA, USA). Platinum Taq DNA Polymerase, SuperScript™ II Reverse Transcriptase, SuperScript III First-Strand Synthesis SuperMix, dNTP Set, TE buffer, GlutaMAX Supplement, 25 and 75 mL polystyrene culture flasks, 12-, 48-, and 96-well plates, ethanol (95%), sodium hydroxide, boric acid, hydrochloric acid, horse serum, fetal calf serum (FCS), and C-Chip disposable hemocytometers were obtained from Thermo Fisher Scientific (Thermo Fisher Waltham, MA, USA). QuantiTect SYBR Green PCR Kits, RNeasy Mini Kit, QuantiTect Primer Assays, and Custom Multi-Analyte ELISArray Kit were purchased from Qiagen (Hilden, Germany). Eppendorf LoBind 1.5 ml Microcentrifuge Tubes PCR Clean and PCR tubes were both supplied by Eppendorf (Hamburg, Germany). Polyethersulfone membrane (3 kDa) centrifuge filters were purchased from VWR International (West Chester, PA, USA). The specific inhibitor of type1 TGF-β1 receptor 4-[4-(1,3-benzodioxol-5-yl)-5-(2-pyridinyl)-1Himidazol-2-yl]benzamide (SB431542) was obtained from the R&D system (Minneapolis, MN, USA). Polydimethylsiloxane (PDMS) microdevices were prepared from a Sylgard 184 elastomer kit (Ellsworth Adhesives, Germantown, WI, USA). Highest Grade Mica Sheets V1 were purchased from Ted Pella Inc (Redding, CA, USA). All water used was ultrapure (18.3 MΩ cm) (Milli-Q Synthesis A10, Millipore, Burlington, MA, USA).

### 2.2. Preparation of Aβ1-42 Oligomers

Aβ1-42 oligomers (oAβ1-42) were prepared as previously described in details elsewhere [46]. In brief, the lyophilized HFIP-treated Aβ1-42 monomers were first suspended in DMSO and then diluted in an ice-cold cell culture medium DMEM/F12 (1:1) at the final concentration of 100 μM. Next, the Aβ1-42 samples (100 μM) were incubated in the absence (oAβ1-42) or presence (Aβ1-42 + Car (co-inc.)) of carnosine at the final concentration of 1 mM for 72 h at 4 °C. After this incubation step, the two (without or with carnosine) Aβ1-42 samples were immediately used or aliquoted and stored at −20 °C until use.

### 2.3. Atomic Force Microscope (AFM)

Amyloid oligomer formation was verified by AFM (Supplementary Figure S1A).

AFM images were collected by using dynamic scanning force microscopy in the air, using a WITec ALPHA300 RS Confocal Raman AFM combined microscope (LOT-QuantumDesign GmbH, Darmstadt, Germany) and etched-silicon probes (Nanosensors, Neuchâtel, Switzerland) with a pyramidal-shaped tip having a radius of curvature <10 nm and a nominal internal angle of 35°. A total of 5 μL of each individual Aβ1-42 sample (oAβ1-42 or Aβ1-42 + Car (co-inc.)) were adsorbed onto the mica and analyzed directly by sensing the adsorbed material with a microfabricated silicon tip attached to a sensitive cantilever. The resulting relief map was subsequently converted into a visual image.

### 2.4. Cell Culture and Preparation

#### 2.4.1. BV-2 Cells

BV-2 cells were cultured in an RPMI-1640 medium enriched with heat-inactivated FBS (10% *v*/*v*), L-glutamine (2 mM), streptomycin (0.3 mg mL^−1^), and penicillin (50 IU mL^−1^). The cells were cultured in 75 mL polystyrene culture flasks at a density of 5 × 10^6^ cells/flask, maintained in a humidified environment at 37 °C and 5% CO_2_/95% air atmosphere, and passaged every 3–5 days depending on the cell confluence in order to avoid cell overgrowth. The day prior to treatment, cells were harvested using a 2.5 mL of trypsin-EDTA solution, counted with a C-Chip disposable hemocytometer, and seeded in 5 mL culture flasks, 12-, or 48-well plates at the appropriate density. Prior to the beginning of each experiment, the exact number of live BV-2 cells necessary for cell seeding was determined by using the trypan blue exclusion assay. For each cell count, 50 μL of cell suspension was diluted 1:2 to 1:5 (based on cell density) with a 0.4% trypan blue solution.

#### 2.4.2. Mixed Neuronal Cultures

Mixed neuronal cultures were obtained from rats at embryonic day 15 (Harlan Laboratories, Italy) as previously described [46,47]. Cells were grown into DMEM/F12 (1:1) and enriched with 10% horse serum, 10% FCS, 2 mM glutamine, and 6 mg/ml glucose. After 7–10 days in vitro, to avoid the proliferation of non-neuronal elements, cytosine-D-arabinoside (at the final concentration of 10 µM) was added to the cultures for 3 days. Cells were then moved into a maintenance serum-free medium. As soon as the right confluence was reached, cells were treated with Aβ oligomers (2 µM) for 48 h both in the presence or in the absence of increasing concentrations of carnosine (1, 5, and 10 mM). The possible neuroprotective activity against Aβ1-42-induced toxicity played by TGF-β1 was indirectly investigated by using the specific inhibitor of type-1 TGF-β receptor, SB431542, at 10 µM as previously accomplished [46].

### 2.5. Measurement of Cell Viability and Cell Death by the MTT and Trypan Blue Exclusion Assays

The effect on the BV-2 cells viability of the treatment with different concentrations (1, 5, and 10 µM) of Aβ1-42 oligomers for 24 h as well as the possible protective effects of carnosine in counteracting Aβ1-42-induced toxicity (Aβ1-42 + Car (co-inc.) or BV-2 cells simultaneously treated with already formed Aβ1-42 oligomers and carnosine (oAβ1-42 + Car (co-treat.)) were measured through the MTT assay as previously reported [48,49]. Briefly, BV-2 cells were seeded in 48-well plates at the density of 1.5 × 10^5^ cells/well. A total of 24 h after cell treatment the medium from each well was removed and the MTT solution (1 mg/mL in RPMI-1640 medium) was added. Following 2 h of incubation at 37 °C and 5% CO_2_/95% air atmosphere, the MTT solution was removed and the formed crystals were dissolved with DMSO. Lastly, 200 µL of each well were transferred to a 96-well plate and the absorbance at 569 nm was read using a plate reader (Spectra Max M5, Molecular Devices, Sunnyvale, CA, USA). Resting (untreated) cells were used as controls.

The toxicity induced in mixed neuronal cultures 48 h after Aβ1-42 oligomers treatment was quantitatively assessed by trypan blue exclusion assay [46,47]. Cell counts were performed in three to four random microscopic fields/well.

### 2.6. NO and O_2_^−•^ Production Determination Using DAF-FM DA and MitoSOX Red Probes

The experiments carried out to investigate the production of NO and O_2_^−•^ were performed as described previously [50] with slight modifications. BV-2 cells previously seeded in 5 mL culture flasks (5 × 10^6^ cells) were treated for 24 h. At the end of the cell treatment, in order to analyze intracellular NO and O_2_^−•^ production, the cells were washed three times with cold PBS (0.01 M, pH 7.4) and then incubated with a phenol red free RPMI-1640 medium containing DAF-FM DA or MitoSOX Red probes previously prepared in 99% sterile DMSO for 1 h. During the incubation time, each flask was covered with aluminum foil to minimize any photobleaching of the probes. Next, the BV-2 cells were harvested, counted, and centrifuged (1137× *g* for 4 min). The obtained cell pellet was washed three times with cold PBS (0.01 M, pH 7.4), lysed using 50 μL of pure ethanol, centrifuged (18.690× *g* for 10 min), and filtered with a polyethersulfone) membrane (3 kDa) centrifuge filter. Then 10 μL of each filtered cell lysate was added to a 90 μL solution consisting of 10 mM boric acid and 7.5 mM SDS at pH 9.2 and transferred to a 96-well plate where the fluorescence was read using a plate reader (Spectra Max M5). Resting cells were used as controls.

In order to detect the real fluorescence due to the reaction between the probes (DAF-FM DA or MitoSOX Red) and the molecules of interest (NO or O_2_^−•^), and to discriminate our compounds from (if any) other fluorescent side products, at least one sample for each experimental condition was run using microchip electrophoresis with laser-induced fluorescence (ME-LIF). The fabrication of PDMS microdevices [51,52], as well as the experimental conditions (sample injection, separation, and detection), data acquisition, and data analysis employed to carry out the ME-LIF experiments, have been described previously [6]. Briefly, a 4’’ diameter silicon wafer was coated with SU-8 10 negative photoresist to a thickness of 15 mm with a Cee 100 spincoater (Brewer Science Inc., Rolla, MO, USA). The obtained wafer was soft baked in two steps (65 °C for 2 min and 95 °C for 5 min) using a programmable hotplate (Thermo Scientific, Asheville, NC, USA). Microchip designs were drawn with AutoCAD (Autodesk Inc., San Rafael, CA, USA) and printed onto a transparency film (Infinite Graphics Inc., Minneapolis MN, USA). The coated wafer was covered with a transparency film mask and exposed to UV light (ABM Inc., San Jose, CA, USA). The wafer was then post-baked in two steps (65 °C for 2 min and 95 °C for 10 min). After the post-bake, the wafer was developed in SU-8 developer, rinsed, and dried. Lastly, the wafer underwent a hard bake at 180–200 °C for 2 h. The final silicon master contained 15 mm thick and 40 mm wide microchannels. In order to complete the final hybrid PDMS-glass microchip device, the PDMS layer was sealed to a borofloat glass plate. Prior to each cell lysate analysis, the PDMS-glass device was flushed with NaOH (0.1 M for 5 min) and a running buffer (10 mM boric acid, 7.5 mM SDS at pH 9.2 for 5 min). Each separation was performed using a 30 kV high voltage power supply (Ultravolt, Ronkonkoma, NY, USA). A total of +2400 V and +2200 V were applied to the running buffer reservoir and sampling reservoir, respectively. The sample was introduced into the separation channel using a 1-s gated injection. To avoid the presence of any residual sample on the channels, the system was flushed for 60 s with a running buffer after each sample analysis. Excitation, detection, data acquisition, and data analysis were carried out using the same technologies and programs already described [6].

A schematic representation of the different steps of the chip manufacturing process, the various components needed for ME-LIF experiments, as well as a representative electropherogram, obtained running a cell sample lysate for NO and O_2_^−•^ detection, are shown in Supplementary Figure S2.

### 2.7. Gene Expression Analysis by Quantitative Real-Time PCR (qRT-PCR)

The total RNA was extracted using the commercial RNeasy Mini Kit according to the manufacturer’s recommendations. The concentration of total RNA recovered from 3.5 × 10^5^ cells (previously seeded in 12-well plates) treated for 6 h was determined by measuring the absorbance at 260 nm with a Varioskan® Flash spectrophotometer (Thermo Fisher Scientific, Waltham, MA, USA). Reverse transcription was performed using 100 ng of total RNA, RNaseH reverse transcriptase, and random primer hexamers (Superscript II, Thermo Fisher Scientific). Next, each sample was quantified, diluted to a final concentration of 25 ng/µL, and used for qRT-PCR analysis (LightCycler® 480 System, Roche Molecular Systems, Inc., Pleasanton, CA, USA). The QuantiTect Primer Assays (Qiagen) employed for the gene expression analysis along with the official name, official symbol, alternative titles/symbols, detected transcript, amplicon length, and primers catalog number are shown in Table 1.

qRT-PCR amplifications were performed in quadruplicate using a mixture of SYBR Green PCR Master Mix (Thermo Fisher Scientific), cDNA samples (100 ng), and specific primers (total reaction volume of 10 μL). Amplification conditions included a first cycle at 95 °C (10 min) followed by 50 cycles at 95 °C (10 seconds), and a final cycle at 60 °C (30 seconds). As a negative control, a reaction in the absence of cDNA (no template control, NTC) was performed and verified by using an Agilent Bioanalyzer 2100 with Agilent DNA 1000 Kit. The relative RNA expression level for each sample was calculated using the 2^−ΔΔCT^ method [53,54] by comparing the threshold cycle (CT) value of the gene of interest to the CT value of our selected internal control (GAPDH gene).

### 2.8. Cytokine Secretion

Cytokines quantification in cell culture supernatants was carried out by using a Custom Multi-Analyte ELISArray Kit according to the manufacturer’s instructions. Briefly, BV-2 cells previously seeded in 48-well plates at the density of 1.5 × 10^5^ cells/well were treated for 24 h and the supernatant from each well was collected, centrifuged at 1000× *g* for 10 min in order to remove any particulate material, and assayed immediately or stored at −80 °C. A total of 50 µL of assay buffer and 50 µL of samples or control samples were added into the appropriate wells of the ELISArray plate and incubated for 2 h at room temperature (RT). After washing 3 times with Wash Buffer, 100 µL of Detection Antibody Solution was added to each well pursued by the following steps: 1 h incubation, 3 washes, the addition of 100 µL Avidin-HRP Conjugate, 30 min incubation at RT, 4 washes, the addition of a 100 µL Development Solution, and 15 min incubation at RT under the dark. As a final step, 100 µL of Stop Solution was added to each well and the absorbance at 450 nm was read using a Synergy H1 Hybrid Multi-Mode Microplate Reader (Biotek, Shoreline, WA, USA) within 30 min of stopping the reaction. As suggested by the vendor, in order to detect the real absorbance, wavelength correction was applied, subtracting the readings at 570 nm from the reading at 450 nm.

### 2.9. Statistical Analysis

Statistical analysis was performed using GraphPad Prism (GraphPad software, San Diego, CA, USA). The within-group comparison was performed by the one-way analysis of variance (ANOVA). The *post hoc* Tukey test was used for multiple comparisons.

### 2.10. Study Approval

The study in mixed neuronal cultures was authorized by the Institutional Animal Care and Use Committee (IACUC) of the University of Catania (OPBA Project #169/2015). Animal care followed Italian (D.M.116192) and EEC (O.J. of E.C. L 358/1 12/18/1986) regulations on the protection of animals used for experimental and scientific purposes.

## 3. Results

### 3.1. Carnosine Protects BV-2 Cells Against Aβ1-42 Oligomers-Induced Cell Death

**Figure 1:**
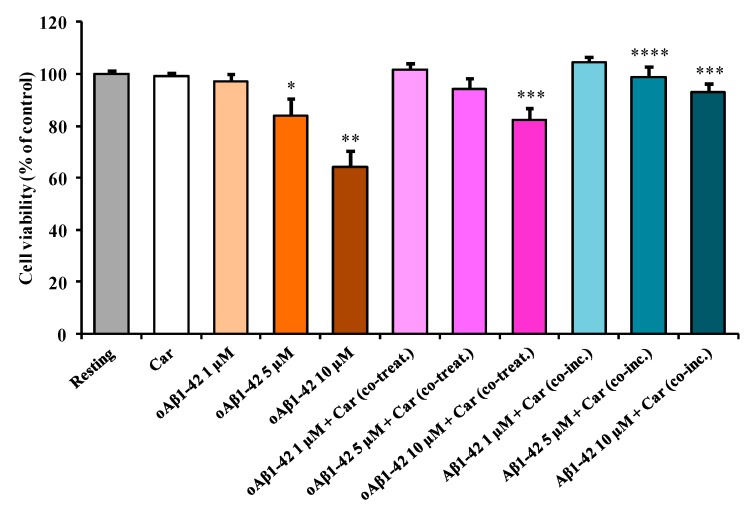
The change in the cell viability caused by challenging BV-2 cells with oAβ1-42 and the protective effects of carnosine. BV-2 cells were treated for 24 h with increasing oAβ1-42 concentrations (1, 5, or 10 µM), in the absence or presence of carnosine (Car, 1 mM) (oAβ1-42 + Car (co-treat.), or with a solution consisting of Aβ1-42 monomers previously incubated in the presence of carnosine (1 mM) during the oligomerization process (Aβ1-42 + Car (co-inc.)); for more details see “Material and Methods” section. Data are the mean (*n* = 4) of 5 independent experiments and are expressed as the percent variation with respect to the viability recorded in resting (control) cells. Standard deviations are represented by vertical bars. * Significantly different from resting cells, *p* < 0.01, ** significantly different from resting cells, *p* < 0.001, *** significantly different from corresponding treatment with no carnosine, *p* < 0.001, **** significantly different from corresponding treatment with no carnosine, *p* < 0.01.

The first aim of the present study was to evaluate the toxicity induced by increasing concentrations of Aβ1-42 oligomers (oAβ1-42) (1, 5, and 10 µM) on microglial BV-2 cells. Data illustrated in Figure 1 show that the treatment of BV-2 cells for 24 h with increasing concentrations of oAβ1-42 provoked a dose-dependent decrease in cell viability.

Unlike 1 µM oAβ1-42 (−3% in cell viability, not significant), the treatment with 5 µM oAβ1-42 led to a significant toxic effect (−19% in cell viability, *p* < 0.01 compared to the resting cells). As expected, the stronger decrease in cell viability (−36%, *p* < 0.001 compared to the resting cells) was observed after the treatment with 10 µM oAβ1-42. In order to examine the protective effects of carnosine, BV-2 cells were treated simultaneously with oAβ1-42 and carnosine (oAβ1-42 + Car (co-treat.)). Figure 1 clearly shows that the BV-2 cells’ viability significantly increased in the presence of carnosine when compared to treatment with the increasing concentrations of oAβ1-42 (1, 5, and 10 µM). A maximal protective effect was observed for cells treated simultaneously with oAβ1-42 10 µM and carnosine (+18% in cell viability, *p* < 0.001 compared to the corresponding treatment with no carnosine). As a part of our toxicity studies, we also challenged BV-2 cells with a solution consisting of Aβ1-42 monomers previously incubated with carnosine during the oligomerization process (Aβ1-42 + Car (co-inc.)). This set of experiments was purposely designed in order to determine whether the well-know anti-aggregation property of carnosine contributed to increasing the cell viability counteracting oAβ1-42 formation and then preventing Aβ toxicity. Considering the presence of carnosine during the oligomerization process, both treatments 5 µM Aβ1-42 + Car (co-inc.) and 10 µM Aβ1-42 + Car (co-inc.) showed cell viability values significantly higher (+15%, *p* < 0.01 and + 29%, *p* < 0.001, respectively) compared to the corresponding treatment with no carnosine.

### 3.2. Carnosine Decreases Aβ1-42-Induced NO Production in Cultured Microglial Cells

**Figure 2:**
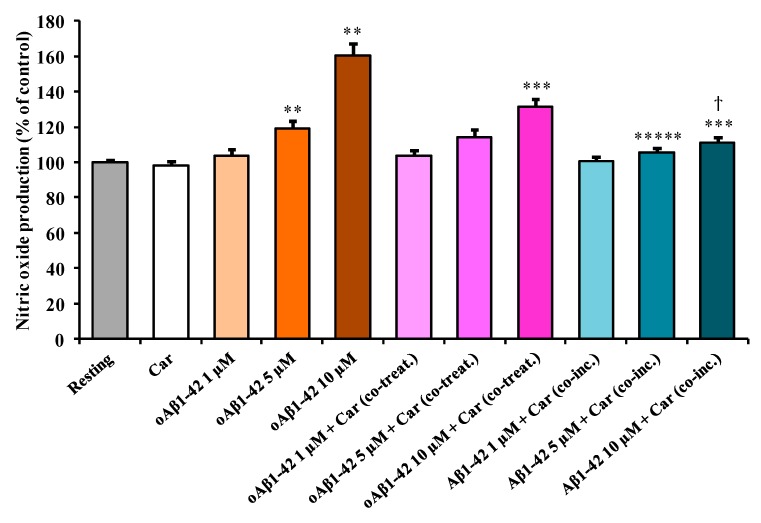
The change in the NO production induced by treating BV-2 cells with Aβ1-42 and effects of carnosine. BV-2 cells were treated for 24 h with increasing oAβ1-42 concentrations (1, 5, or 10 µM), in the absence or presence of carnosine (Car, 1 mM) (oAβ1-42 + Car (co-treat.), or with a solution consisting of Aβ1-42 monomers previously incubated in the presence of carnosine (1 mM) during the oligomerization process (Aβ1-42 + Car (co-inc.)). Data are the mean of 5 independent experiments and are expressed as the percent variation with respect to the NO production recorded in resting cells. Standard deviations are represented by vertical bars. ** Significantly different from resting cells, *p* < 0.001, *** significantly different from corresponding treatment with no carnosine, *p* < 0.001, ***** significantly different from corresponding treatment with no carnosine, *p* < 0.05, ^ϯ^ significantly different from cells treated with 10 µM oAβ1-42 + Car (co-treat), *p* < 0.001.

Figure 2 shows the effect of Aβ1-42 treatment on the intracellular NO production in BV-2 cells.

This increase in NO production was significant in the case of both 5 µM oAβ1-42 (+19%, *p* < 0.001 compared to resting cells) and 10 µM oAβ1-42 (+60%, *p* < 0.001 compared to resting cells) treatments. The addition of carnosine to the resting BV-2 cells did not cause any significant change in the basal microglia NO production. To test the effect of carnosine on NO production in stimulated BV-2 cells, carnosine was added along with three different concentrations of oAβ1-42. The amount of NO production was essentially the same for cells treated with 1 µM oAβ1-42, in the presence or absence of carnosine. A slight, but not significant, decrease (−5%) was measured in 5 µM oAβ1-42 + Car (co-treat.) compared to cells stimulated in the absence of carnosine. The production due to the treatment with 10 µM oAβ1-42 was significantly lowered by the presence of carnosine (−29%, *p* < 0.001). As for 1 µM oAβ1-42 + Car (co-treat.), the production of NO for Aβ1-42 1 µM + Car (co-inc.) treatment was comparable to the one detected in the resting cells. The presence of carnosine during the oligomerization process strongly decreased the effect of Aβ1-42 in inducing NO production. In fact, both 5 µM Aβ1-42 + Car (co-inc.) (−14%, *p* < 0.05) and 10 µM Aβ1-42 + Car (co-inc.) (−49%, *p* < 0.001) treatments showed a significant decrease in NO production when compared with the corresponding treatment in the absence of carnosine. Interestingly, the production of NO for the 10 µM Aβ1-42 + Car (co-inc.) treatment was significantly lower than 10 µM oAβ1-42 + Car (co-treat.) (−20 %, *p* < 0.001).

### 3.3. Carnosine Decreases Aβ1-42-Induced O_2_^−•^ Production in Cultured Microglial Cells

**Figure 3:**
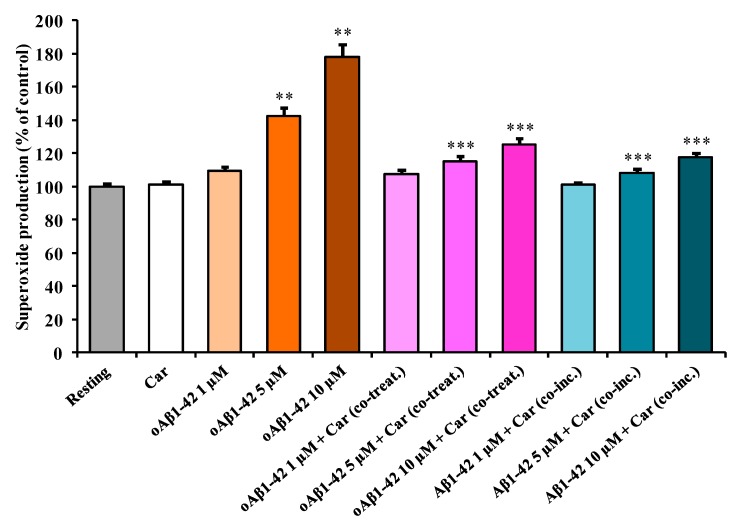
The change in O_2_^−•^ production induced by treating BV-2 cells with Aβ1-42 and effects of carnosine. BV-2 cells were treated for 24 h with increasing oAβ1-42 concentrations (1, 5, or 10 µM), in the absence or presence of carnosine (Car, 1 mM) (oAβ1-42 + Car (co-treat.), or with a solution consisting of Aβ1-42 monomers previously incubated in the presence of carnosine (1 mM) during the oligomerization process (Aβ1-42 + Car (co-inc.)). Data are the mean of 5 independent experiments and are expressed as the percent variation with respect to the nitric oxide production recorded in resting cells. Standard deviations are represented by vertical bars. ** Significantly different from resting cells, *p* < 0.001, *** significantly different from corresponding treatment with no carnosine, *p* < 0.001.

Figure 3 depicts the effect of Aβ1-42 treatment on the intracellular O_2_^−•^ production in BV-2 cells.

As observed in the case of NO production, the stimulation of the cells with increasing concentration of oAβ1-42 caused a dose-dependent increase in O_2_^−•^ production. This increase was significant in the case of both 5 µM oAβ1-42 (+43%, *p* < 0.001 compared to resting cells) and 10 µM oAβ1-42 (+78%, *p* < 0.001 compared to resting cells) treatments. A slight, but not significant, increase (+9%) compared to resting cells was measured in cells treated with 1 µM oAβ1-42. The addition of carnosine to resting BV-2 cells did not cause any significant change in the basal microglia O_2_^−•^ production. To test the effect of carnosine on O_2_^−•^ production in stimulated BV-2 cells, carnosine was added along with three different concentrations of oAβ1-42. The amount of O_2_^−•^ production was essentially the same for cells treated with 1 µM oAβ1-42, in the presence or absence of carnosine. The co-treatment with carnosine was able to counteract O_2_^−•^ production in cell stimulated with 5 µM oAβ1-42 (−29%, *p* < 0.001) as well as 10 µM oAβ1-42 (−53%, *p* < 0.001). As for 1 µM oAβ1-42 + Car (co-treat.), the production of O_2_^−•^ was essentially the same of the controls for Aβ1-42 1 µM + Car (co-inc.). Once again, as previously observed for NO production, the presence of carnosine during the oligomerization process strongly decreased the effects of Aβ1-42 in inducing O_2_^−•^ production. In fact, both 5 µM Aβ1-42 + Car (co-inc.) (−34%, *p* < 0.001) and 10 µM Aβ1-42 + Car (co-inc.) (–60%, *p* < 0.001) treatments showed a significant decrease in O_2_^−•^ production when compared with the corresponding treatment in the absence of carnosine. A slight, but not significant, decrease (−9%) was measured for 1 µM Aβ1-42 + Car (co-inc.) compared to the 1 µM oAβ1-42 treatment.

It is also worth underlining that in our experimental model the treatment of BV-2 cells with oAβ1-42 produced more evident effects on O_2_^−•^ production than those detected for NO production, at all concentrations (1, 5, or 10 µM) tested.

By combining the information obtained from the first three sets of experiments (Figure 1, Figure 2 and Figure 3), we selected the optimal oAβ1-42 concentration (10 µM) able to generate the strongest response in BV-2 cells, then used it, in the absence or presence of carnosine, to analyze mRNA expression and protein secretion.

### 3.4. Carnosine Decreases Aβ1-42-Induced mRNA Expression Level of iNOS, Nox1, and Nox2 and Increases TGF-β1 mRNA Expression in Cultured Microglial Cells

**Figure 4:**
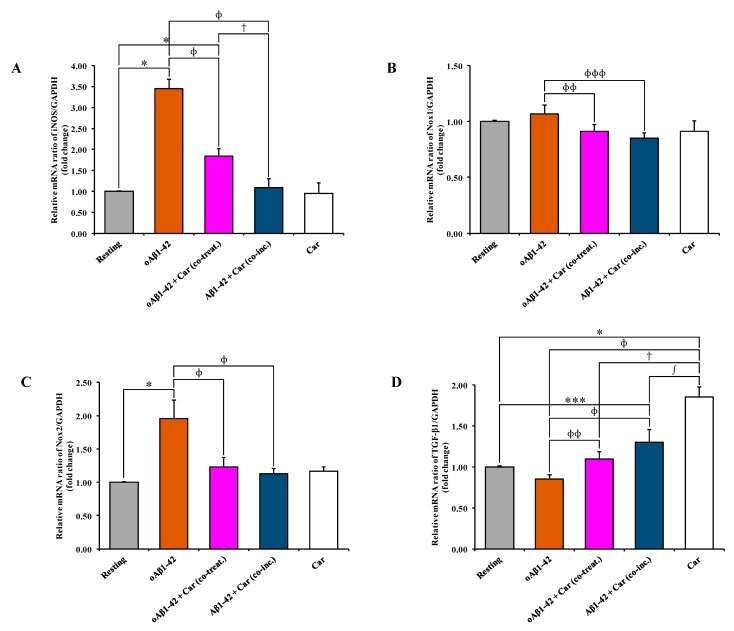
Carnosine suppresses the Aβ1-42-induced mRNA expression levels of iNOS, Nox1, and Nox2 and increases the expression of TGF-β1 mRNA. Effects of Aβ1-42 and carnosine (Aβ1-42 + Car (co-treat.) and Aβ1-42 + Car (co-inc.)) on (**A**) iNOS, (**B**) Nox1, (**C**) Nox2, and (**D**) TGF-β1 mRNAs expression were examined by qRT-PCR. The abundance of each mRNA of interest was expressed relative to the abundance of GAPDH-mRNA, as an internal control. As a negative control, a reaction in the absence of cDNA (no template control, NTC) was performed. qRT-PCR amplifications were performed in quadruplicate. Standard deviations are represented by vertical bars. * significantly different from resting cells, *p* < 0.001, ** significantly different from resting cells, *p* < 0.05, *** significantly different from resting cells, *p* < 0.01, ^ɸ^ significantly different from oAβ1-42-treated cells, *p* < 0.001, ^ɸɸ^ significantly different from oAβ1-42-treated cells, *p* < 0.05, ^ɸɸɸ^ significantly different from oAβ1-42-treated cells, *p* < 0.01, ^ϯ^ significantly different from oAβ1-42 + Car (co-treat.)-treated cells, *p* < 0.001, ^∫^ significantly different from Aβ1-42 + Car (co-inc.)-treated cells, *p* < 0.001.

Since the treatment of BV-2 cells with carnosine decreased the oAβ1-42-induced production of NO and O_2_^−•^ (Figure 2 and Figure 3, respectively), we assessed the ability of carnosine to modulate the expression of iNOS and Nox subunits as well as the synthesis and the release of several cytokines related to Aβ-induced inflammation in microglial cells. As expected, the expression level of iNOS mRNA was significantly increased (3.45 folds) following oAβ1-42 treatment (*p* < 0.001 compared to resting cells) (Figure 4A).

Both oAβ1-42 + Car (co-treat.) and Aβ1-42 + Car (co-inc.) treatments were able to counteract Aβ1-42-induced iNOS activation (*p* < 0.001 compared to oAβ1-42-treated cells). The strongest inhibitory effect was observed for the Aβ1-42 + Car (co-inc.) treatment (from 3.45 folds to 1.09 folds) compared to the simple co-treatment with carnosine. The addition of carnosine to resting BV-2 cells did not cause any significant change in the expression level of iNOS mRNA. A slight, but not significant, increase (1.07 folds) was measured for Nox1 mRNA following oAβ1-42 treatment, while values lower than the control were observed for oAβ1-42 + Car (co-treat.) (0.91 folds) and Aβ1-42 + Car (co-inc.) (0.87 folds) treatments (Figure 4B). As observed for iNOS mRNA expression, the stimulation of BV-2 cells with oAβ1-42 significantly increased the expression of Nox2 mRNA (1.96 folds, *p* < 0.001) (Figure 4C). Both oAβ1-42 + Car (co-treat.) and Aβ1-42 + Car (co-inc.) treatments gave values significantly lower than cells treated with Aβ1-42 in the absence of carnosine (*p* < 0.001 compared to oAβ1-42). Figure 4D shows the effect of Aβ1-42 treatment, alone or in combination with carnosine, on the expression level of TGF-β1 mRNA in BV-2 cells. A slight, but not significant, decrease (0.85 folds) was measured for TGF-β1 mRNA following oAβ1-42 treatment. The co-treatment with carnosine increased the expression level to 1.09 folds (*p* < 0.05 compared to oAβ1-42-treated cells). For Aβ1-42 + Car (co-inc.) treatment the expression of TGF-β1 measured was equal to 1.30 folds (*p* < 0.01 compared to resting cells; *p* < 0.001 compared to oAβ1-42-treated cells). Interestingly, carnosine per se provoked a significant increase (1.85 folds) in the expression level of TGF-β1 mRNA in resting BV-2 cells (*p* < 0.001 compared to all other treatments). At this time point (6 h), the expression level of IL-6 mRNA in BV-2 cells did not significantly change in all the experimental conditions (data not shown).

### 3.5. Carnosine Modulates the Release of Pro- and Anti-Inflammatory Cytokines in Cultured Microglial Cells

**Figure 5:**
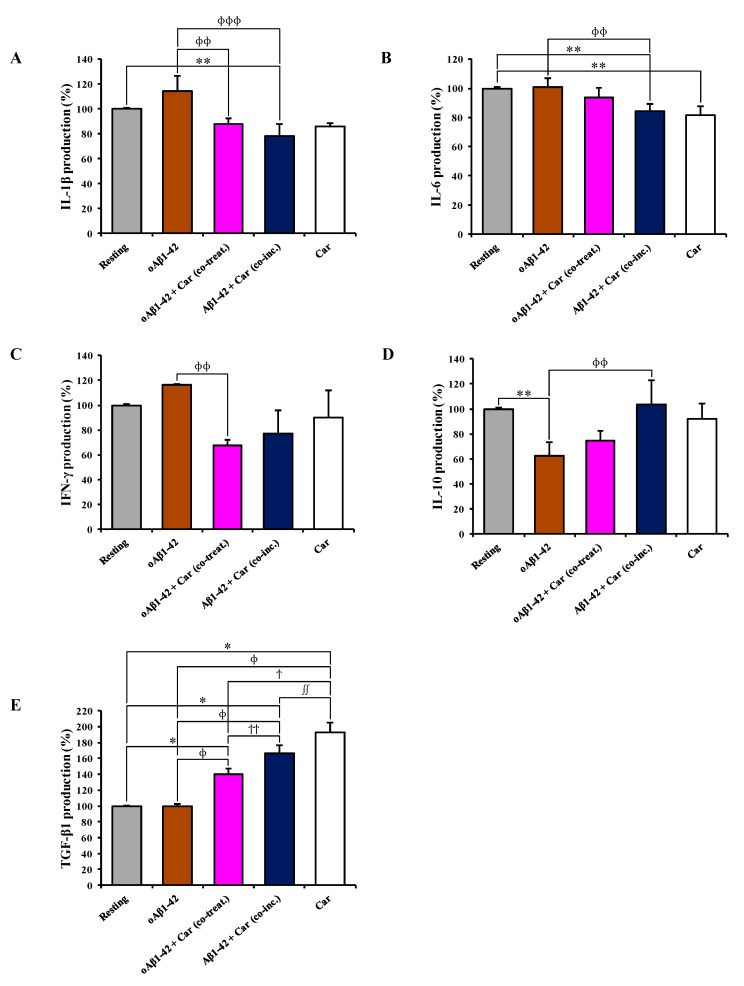
The modulation of cytokines secretion by carnosine. Supernatants from resting and BV-2 cells stimulated with oAβ1-42 in the absence or presence of carnosine (oAβ1-42 + Car (co-treat.) and Aβ1-42 + Car (co-inc.)) were analyzed using a Custom Multi-Analyte ELISArray Kit. Each treatment was analyzed in triplicate. The production of each cytokine is expressed as the percent variation with respect to the production recorded in resting cells. (**A**) IL-1β, (**B**) IL-6, (**C**) IFN-γ, (**D**) IL-10, and **E**) TGF-β1. Standard deviations are represented by vertical bars. * Significantly different from resting cells, *p* < 0.001, ** significantly different from resting cells, *p* < 0.05, ^ɸ^ significantly different from oAβ1-42-treated cells, *p* < 0.001, ^ɸɸ^ significantly different from oAβ1-42-treated cells, *p* < 0.05, ^ɸɸɸ^ significantly different from oAβ1-42-treated cells, *p* < 0.01, ^ϯ^ significantly different from oAβ1-42 + Car (co-treat.)-treated cells, *p* < 0.001, ^ϯϯ^ significantly different from oAβ1-42 + Car (co-treat.)-treated cells, *p* < 0.05, ^∫^ significantly different from Aβ1-42 + Car (co-inc.)-treated cells, *p* < 0.001, ^∫∫^ significantly different from Aβ1-42 + Car (co-inc.)-treated cells, *p* < 0.05.

The analysis of cytokines in cell supernatants indicated an up-regulation (+15%) of the pro-inflammatory cytokine IL-1β induced by oAβ1-42 treatment compared to resting cells (Figure 5A).

The presence of carnosine along with Aβ1-42 down-regulated the IL-1β production, with Aβ1-42 + Car (co-inc.) having a stronger effect (–36%, *p* < 0.001 compared to oAβ1-42-treated cells) than that of oAβ1-42 + Car (co-treat.) (–27%, *p* < 0.05 compared to oAβ1-42-treated cells). The addition of carnosine to resting BV-2 cells did not cause any significant change in the release of IL-1β. The treatment of BV-2 cells with oAβ1-42, alone or in co-treatment with carnosine, did not lead to any significant change in the production of the pro-inflammatory cytokine IL-6 (Figure 5B). On the contrary, both Aβ1-42 + Car (co-inc.) and carnosine alone treatments down-regulated IL-6 production (*p* < 0.05 compared to resting and oAβ1-42-treated cells and *p* < 0.05 compared to resting, respectively). oAβ1-42 treatment up-regulated IFN-γ (+16%) in BV-2 cells (Figure 5C). Both treatments with carnosine down-regulated the IFN-γ production compared to the corresponding treatment with no carnosine, with oAβ1-42 + Car (co-treat.) treatment showing a stronger and significant effect (−33%, *p* < 0.05 compared to oAβ1-42-treated cells) than that of Aβ1-42 + Car (co-inc.) (−23%). The treatment with carnosine alone lowered IFN-γ by 10%. Figure 5D,E shows the modulation in the release of the two major anti-inflammatory cytokines (IL-10 and TGF-β1) by Aβ1-42 in the absence or presence of carnosine. oAβ1-42 treatment strongly down-regulated IL-10 production (−38%, *p* < 0.05 compared to resting cells). oAβ1-42 + Car (co-treat.) treatment slightly increased the production of IL-10 (+12%) while Aβ1-42 + Car (co-inc.) treatment rescued the IL-10 release to the values detected in resting cells, significantly higher than that of oAβ1-42 alone (+41%, *p* < 0.05) (Figure 5D). As in the case of IL-1β, the addition of carnosine to resting BV-2 cells did not cause any significant change in the release of IL-10. The level of TGF-β1 was very similar between resting and oAβ1-42-treated BV-2 cells (Figure 5E). The treatment with carnosine along with oAβ1-42 markedly up-regulated TGF-β1 (+40%, *p* < 0.001 compared to resting and oAβ1-42-treated cells) while the Aβ1-42 + Car (co-inc.) treatment led to an increase of TGF-β1 production equal to +66% compared to resting and oAβ1-42-treated cells (*p* < 0.001) and +26% compared to oAβ1-42 + Car (co-treat.) treatment (*p* < 0.05). Once again, as already observed for the expression level of TGF-β1 mRNA, the addition of carnosine per se to resting BV-2 cells strongly up-regulated the release of TGF-β1 (+93%, *p* < 0.05 compared to Aβ1-42 + Car (co-inc.)-treated cells, *p* < 0.001 compared to all other treatments).

### 3.6. Carnosine Prevents oAβ1-42-Induced Toxicity in Mixed Neuronal Cultures via TGF-β1

Finally, we examined the neuroprotective activity of carnosine in mixed neuronal cultures containing both neurons (35–40%) and glial cells (astrocytes and microglia; 60–65%) challenged with oAβ1-42 (2 µM). We have previously demonstrated that mixed neuronal cultures treated with oAβ1-42 represent an established experimental model of Aβ-induced neurodegeneration, where oAβ1-42 show a faster kinetics compared to pure neuronal cultures, with a substantial increase in the number of dead neurons (about 100%) being detected after 48 h of exposure to Aβ oligomers [55].

**Figure 6:**
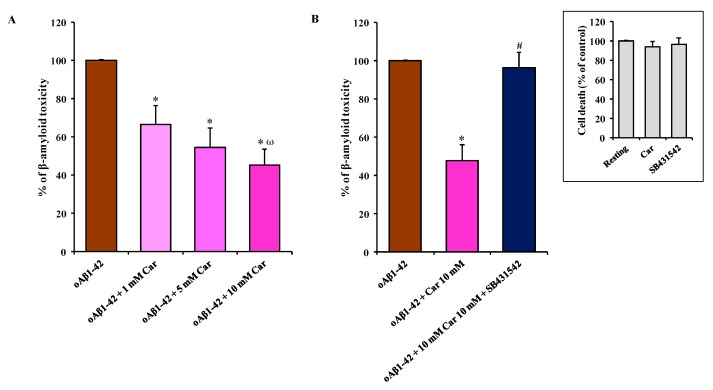
The neuroprotective effects of carnosine against oAβ1-42-induced toxicity are mediated by TGF-β1. (**A**) Mixed neuronal cultures were challenged with oAβ1-42 (2 µM) for 48 h in the absence or presence of increasing concentrations of carnosine (1, 5, and 10 mM). (**B**) Effect of SB431542 (specific inhibitor of type-1 TGF-β receptor) treatment (10 µM) on the neuroprotective activity of carnosine against oAβ1-42-induced toxicity. oAβ1-42 toxicity in mixed neuronal cultures was assessed by cell counting after trypan blue staining. Cell counts were performed in three to four random microscopic fields/well. Data are the mean of 6 (A) or 5 (B) determinations and are expressed as the percent variation with respect to the cell death recorded in oAβ1-42-treated cells. Standard deviations are represented by vertical bars. * Significantly different from oAβ1-42-treated cells, *p* < 0.001, ^ω^ significantly different from oAβ1-42 + Car 1 mM, *p* < 0.001, # significantly different from oAβ1-42 + Car 10 mM.

Figure 6A clearly shows the neuroprotective effects of carnosine against oAβ1-42-induced toxicity.

Carnosine decreased the oAβ1-42 toxic effect in mixed neuronal cultures in a dose-dependent manner, with 10 mM carnosine (oAβ1-42 + 10 mM Car) having a stronger protective effect (-55% in cell death, *p* < 0.001 compared to oAβ1-42 and oAβ1-42 + 1 mM Car). The presence of carnosine at the final concentration of 1 or 5 mM reduced oAβ1-42-induced cell death by 34% and 46%, respectively (*p* < 0.001 compared to oAβ1-42-treated cells). As shown in Figure 6B, the specific inhibitor of type-1 TGF-β receptor, SB431542, prevented the neuroprotective activity of carnosine that was directly applied to mixed neuronal cultures challenged with oAβ1-42 (*p* < 0.001 compared to oAβ1-42 + 10 mM Car) suggesting that TGF-β1 release and activation of TGF-β1 signaling play a central role in mediating the neuroprotective effects of carnosine. The percentage of toxicity due to the presence of oAβ1-42 decreased by 53% (47% of cell death) in the presence of carnosine, but it increased again to a value very similar to oAβ1-42 treatment in the presence of SB431542 (96% of cell death). The treatment with SB431542 (10 µM) as well as with carnosine (10 mM) alone had no significant effects per se on neuronal death in mixed neuronal cultures in the absence of oAβ1-42 treatment (Figure 6B, insert).

## 4. Discussion

Oligomers, the most toxic species of Aβ1-42 aggregated forms, cause neuronal dysfunction and death in AD brains [56]. Microglial cells and neuronal cultures challenged with synthetic analogs of human oligomers of Aβ1-42 provide a widely accepted and reliable model of neuroinflammation and neurodegeneration occurring in AD [47,57,58]. In this scenario, oxidative stress plays a central role in Aβ-induced neurodegeneration [59,60].

Oxidative stress is a process referring to an imbalance between pro-oxidants, such as ROS and RNS, and antioxidants in favor of pro-oxidants. A wide body of literature supports the negative impact and key role played by this phenomenon in the pathogenesis of AD [61], preceding the appearance of the two hallmarks of this disease represented by the abnormal deposition of Aβ (senile plaques) and the intracellular accumulation of hyperphosphorylated tau protein (neurofibrillary tangles) [62]. In particular, on one hand the oligomeric form of Aβ peptide impairs synaptic plasticity, and promotes neurodegeneration and neuroinflammation through oxidative stress [63,64]; on the other hand oxidative stress favors Aβ oligomerization [65].

In the present study, we first explored the toxicity induced by different concentrations (1, 5, and 10 µM) of Aβ1-42 oligomers on BV-2 microglial cells and then examined the correlation between Aβ toxicity and the production of NO and O_2_^−•^, two well-known reactive species that significantly contribute to neurodegeneration in AD [66,67]. This order of magnitude (µM) is physiologically relevant since from one hand under normal physiological conditions and in AD patients the concentration of Aβ peptide in brain extracellular fluid is low (pM to nM levels) [68,69,70]; on the other hand in vitro studies suggest that the critical concentration for spontaneous aggregation (e.g., oligomer formation) of Aβ peptide is in the µM range [71,72]. Accordingly, Aβ concentrations in vivo would have to increase by at least 3 to 4 orders of magnitude (e.g., close to the amyloid plaques) for spontaneous aggregation to be feasible in the extracellular space. When monitoring the change in cell viability, we observed that oAβ1-42 decreased BV-2 cell viability in a dose-dependent manner (Figure 1). BV-2 cells were able to counteract amyloid-induced cell toxicity only at low concentrations (1 or 5 µM) while at the highest Aβ concentration (10 µM) the well-known ability of microglia in amyloid clearance [73] was overcome by Aβ toxicity. Figure 1 also shows the protective effects of carnosine co-treatment in all the conditions tested. We hypothesized that these protective effects could be due to the ability of carnosine in counteracting both oxidative stress and inflammation in microglial cells [52,74]. In fact, as showed by Fleisher-Berkovich et al., carnosine as well as its acetylated form are able to decrease LPS-induced microglial oxidative stress and inflammation [74]. Furthermore, carnosine has been shown to protect neurons against oxidative stress by modulating the MAPK cascade signaling [3]. The neuroprotective effects exerted by carnosine have also been demonstrated by Lopachev et al. by using the primary culture of rat cerebellar cells under oxidative stress [75]. Our hypothesis was also strongly corroborated by the direct measurement of NO and O_2_^−•^ intracellular levels as well as the expression of iNOS and Nox enzymes in Aβ-stimulated cells. The levels of these reactive species increased in a dose-dependent manner by Aβ1-42 oligomers, whereas they significantly diminished in the presence of carnosine (Figure 2 and Figure 3) in accordance to its antioxidant activity and the ability of this peptide to directly interact with these species, decreasing their availability [6,76]. In particular, part of the observed decreased toxicity could be due to an increased uptake of carnosine by immune cells under stress conditions [77], to the ability of this dipeptide to convert NO into its not-toxic end-product nitrite [6], and/or carnosine capability to disassemble aggregate structures already formed [78,79]. In accordance to the viability and NO and O_2_^−•^ results, Figure 4A–C shows that the decrease in reactive species depends not only on the scavenging activity of carnosine [80] but also on the ability of this peptide to decrease the expression Aβ-induced enzymes related to oxidative and nitrosative stress.

As a part of our study, instead of co-treating with carnosine and Aβ1-42 oligomers already formed, we challenged BV-2 cells with a solution consisting of Aβ1-42 monomers previously incubated in the presence of carnosine during the oligomerization process (indicated in each figure as Aβ1-42 + Car. (co-inc.)). Our aim was to determine whether the well-known anti-aggregation property of carnosine [81,82,83] contributed to decreasing the cell toxicity and oxidative stress by preventing the formation of Aβ1-42 toxic species. As expected, carnosine, by inhibiting oligomers formation (confirmed by AFM analysis (Supplementary Figure S1B), protected microglial cells (Figure 1), reduced NO (Figure 2) and O_2_^−•^ (Figure 3) intracellular levels, and inhibited iNOS and Nox up-regulation (Figure 4A–C). These results are in accordance with Corona et al. which showed that carnosine supplementation in 3xTg-AD mice, a transgenic model of AD, led to a strong reduction in the hippocampal intraneuronal accumulation of Aβ, completely rescuing AD and aging-related mitochondrial dysfunctions [4]. It is worth underlining that when considering the different protective effects observed between oAβ1-42 + Car. (co-treat.) and Aβ1-42 + Car. (co-inc.) treatments, the latter gave always slightly stronger effects. The increased protective effect observed with carnosine in microglial cells after the co-incubation of Aβ monomers with a millimolar concentration of carnosine suggests that the anti-aggregation properties of carnosine [5,78,79] significantly contribute to increase the overall protective effects of carnosine against Aβ1-42 toxicity in addition to the antioxidant activity of this peptide. Furthermore, our data obtained with AFM suggest that carnosine might preserve Aβ monomers, which are essential for neuronal survival and maintaining neuronal glucose homeostasis [84,85], and it can also promote the dissociation of Aβ oligomers. Future studies are needed to assess whether carnosine can act as a monomer stabilizer, preventing the transition from Aβ monomers to Aβ oligomers.

Activated microglia and astrocytes are the main source of cytokines in the brain [86], and elevated markers of microglial activation (measured by translocator protein binding in vivo with PET) have been found in AD patients [87]. Aβ oligomers promote neuroinflammation and neurodegeneration in AD brains by eliciting the release of pro-inflammatory cytokines from microglia cells [88].

In the present study, we adopted an experimental model of neuroinflammation, where BV-2 microglial cells were challenged with a micromolar concentration of Aβ oligomers [57,58]. Aβ-induced inflammation in these cells is also known to strictly correlate with oxidative stress and an increase in ROS formation [89,90].

In our experimental model, Aβ oligomers (24 h treatment) significantly reduced the secretion of anti-inflammatory cytokines such as IL-10 (Figure 5D), whereas no statistically significant effect was observed for IL-1β and IFN-γ secretion (Figure 5A,C) as well as for IL-6 and TGF-β1 release (Figure 5B,E). With particular regard to IL-6, it is important to underline that differently from the gene expression analysis (6 h treatment), where the expression level of IL-6 mRNA was not changed by carnosine, cytokine secretion experiments (24 h treatment) demonstrate the ability of carnosine to decrease IL-6 levels. This suggests that under our experimental conditions: i) carnosine may require more than 6 h to directly modulate IL-6 gene expression; ii) carnosine could decrease IL-6 at post-translational level by direct interaction and/or modulating signal transduction pathways connected to its production such as phospholipases C and D [91]. Interestingly carnosine rescued IL-10 levels in Aβ-treated BV-2 cells and also reduced IL-1β, IL-6, and IFN-γ levels as assessed by the ELISA assay (Figure 5). Previous studies conducted in vivo have demonstrated that carnosine reduces both oxidative stress and microglial activation in animal models of subcortical ischemic vascular dementia and subarachnoid hemorrhage model [92,93,94,95], but no studies have been yet conducted in animal models of AD to examine the effects of carnosine on microglia activation. As discussed above, it is known from previous in vitro studies that carnosine can prevent LPS-induced microglial inflammation and oxidative damage [74], but our in vitro study is the first evidence that carnosine can counteract in microglial cells both oxidative stress and the release of pro-inflammatory cytokines induced by Aβ oligomers. Furthermore, carnosine showed the ability to rescue IL-10 levels, an anti-inflammatory cytokine whose deficit in AD patients seems to play a key role in promoting neuroinflammation and cognitive deficits [96]. Future studies are needed to establish whether carnosine can exert this effect on IL-10 production in vivo in animal models of AD.

Interestingly we found that carnosine exerted a specific effect on the expression of TGF-β1, the only cytokine whose mRNA levels were significantly affected by carnosine with a relevant increase at 6 h (Figure 4D) followed by a strong increase in TGF-β1 release at 24 h (Figure 5E). We focused our attention on this anti-inflammatory cytokine because it is well-known that TGF-β1 exerts strong anti-inflammatory and neuroprotective effects in experimental models of AD [97]. It also plays a constitutive role in the suppression of inflammation, controlling the degree of microglial activation in the central nervous system [98] and stimulating Aβ clearance by microglia [99]. It has also been recently demonstrated that microglial activation induced by Aβ1-42 oligomers results in the inhibition of TGF-β-regulated gene expression in primary rat microglia [100]. When considering this evidence it is relevant to note that in our experimental model of Aβ-induced inflammation carnosine was able to promote both the synthesis and the release of TGF-β1 from microglial cells. This effect of carnosine is relevant when taking into account the role of TGF-β1 in the pathophysiology of AD [101]. The selective impairment of the TGF-β1 signaling pathway has been demonstrated in the early phase of AD pathogenesis [102] and this deficit of TGF-β1 contributes to neuroinflammation and cognitive decline in AD [103]. Therefore, the rescue of TGF-β1 signaling represents a new pharmacological strategy to yield neuroprotection in AD and second-generation antidepressants such as fluoxetine increase the release of TGF-β1 from astrocytes and exert relevant neuroprotective effects in experimental models of AD [46].

Starting from this evidence, we examined the neuroprotective effects of carnosine in mixed neuronal cultures challenged with Aβ oligomers, an established an experimental model of Aβ-induced neurodegeneration [46,47]. Interestingly we found that carnosine started to prevent Aβ toxicity at 1 mM, with a further relevant increase of its neuroprotective efficacy at 10 mM (with 55% of neuronal rescue) (Figure 6A). For our knowledge, this is the first evidence that carnosine can prevent Aβ-induced neuronal death in an in vitro model of Aβ-induced neurodegeneration. The protection observed following carnosine treatment was in part expected since our results (Figure 4D and Figure 5D,E) show that this dipeptide is able to enhance the ability of microglial cells to produce anti-inflammatory mediators (e.g., IL-10 and TGF-β1). According to this scenario, it is expected that the high percentage of glial cells (60–65%) in our co-culture model heavily contribute to neuronal protection. The ability of SB431542, a selective inhibitor of the type-1 TGF-β receptor, to completely prevent the effects of carnosine (Figure 6B) suggests that TGF-β1 release and activation of Smad-dependent TGF-β1 signaling play key roles in mediating the neuroprotective efficacy of carnosine against Aβ toxicity. Future studies should be conducted in transgenic animal models of AD to assess whether carnosine can prevent amyloid-related cognitive deficits by the rescue of TGF-β1 signaling.

## 5. Conclusions

In the present study, we reported for the first time that carnosine prevents Aβ-induced oxidative stress in BV-2 microglial cells by decreasing the expression of inducible nitric oxide synthase and NADPH oxidase and the concentrations of nitric oxide and superoxide anion. We demonstrated for the first time that, in an established model of Aβ-induced inflammation, carnosine was able to decrease the secretion of pro-inflammatory cytokines such as IL-1β, simultaneously rescuing IL-10 levels and increasing the synthesis and the release of TGF-β1. We then validated the protective activity of carnosine in mixed neuronal cultures challenged with Aβ1-42 oligomers, where carnosine prevented Aβ-induced neurodegenerative phenomena via the activation of TGF-β1 signaling.

The inhibition of Aβ oligomer-mediated inflammation and rescue of TGF-β1 signaling have been recently considered effective strategies for protecting against neurodegeneration and disease progression in AD. Carnosine, through its multimodal mechanism of action, might represent a new pharmacological tool to yield neuroprotection in AD.

## Figures and Tables

**Table 1:** The list of primers used for quantitative real-time PCR (qRT-PCR).

Official Name ^#^	Official Symbol	Alternative Titles/Symbols	Detected Transcript	Amplicon Length	Cat. No. ^§^
nitric oxide synthase 2, inducible	Nos2	iNOS; Nos-2; Nos2a; i-NOS; NOS-II; MAC-NOS	NM_010927	118 bp	QT00100275
NADPH oxidase 1	Nox1	MOX1; NOH1; NOH-1; NOX1a; Nox-1; GP91-2; NOX1alpha	NM_172203 XM_006528515	180 bp	QT00140091
cytochrome b-245, beta polypeptide	Cybb	Cgd; Cyd; Nox2; C88302; gp91-1; gp91phox; CGD91-phox	NM_007807 XM_006527565	146 bp	QT00139797
transforming growth factor, beta 1	Tgfb1	Tgfb; Tgfb-1; TGFbeta1; TGF-beta1	NM_011577	145 bp	QT00145250
interleukin 6	Il6	Il-6	NM_031168	128 bp	QT00098875
glyceraldehyde-3-phosphate dehydrogenase	Gapdh	Gapd	NM_008084 XM_001003314 XM_990238 NM_001289726	144 bp	QT01658692

^#^https://www.ncbi.nlm.nih.gov/gene/. ^§^https://www.qiagen.com/it/shop/pcr/real-time-pcr-enzymes-and-kits/two-step-qrt-pcr/quantitect-primer-assays/.

## Supplementary Materials

Supplementary materials can be found at http://www.mdpi.com/2073-4409/8/1/64/s1. Figure S1: AFM analysis of Aβ1-42 samples, Figure S2: A schematic representation of chip manufacturing process, ME-LIF setup, and representative electropherograms.
