# Supplementary material for: Carnosine Prevents Aβ-Induced Oxidative Stress and Inflammation in Microglial Cells: A Key Role of TGF-β1
Source: Cells. 2019 Jan 17;8(1):64. doi: 10.3390/cells8010064 (PMC6356400; doi:10.3390/cells8010064)
Supplement: Supplementary file 1 [file cells-08-00064-s001.zip › Supplementary figure legends.pdf]

Supplementary figure legends

Supplementary Figure S1. AFM analysis of Aβ1-42 samples. During the oligomerization process (72 h at 4 °C), Aβ1-42 monomers (100 μM) were incubated in absence or presence of carnosine at the final concentration of 1 mM. A) Phase and height AFM images of an Aβ1-42 sample after the oligomerization process in absence of carnosine (oAβ1-42); B) Height AFM image of an Aβ1-42 sample after the oligomerization process in presence of carnosine (Aβ1-42 + Car (co-inc.)).

Supplementary Figure S2. A schematic representation of chip manufacturing process, ME-LIF setup, and representative electropherograms. I) different steps of chip manufacturing process: a = PDMS (Polydimethylsiloxane) + Curing agent (1:10 ratio); b = Plastic cup and Stick; c = Vacuum pump; d = Master chip; e = Oven (70°C overnight); f = Parafilm and punch holes (4 mm) in the chip. II) ME-LIF setup: Microfluidic chip: simple-T device with a 5 cm separation channel and 0.75 cm side arms; PMT = photomultiplier tubes; III) Representative electropherograms of cell lysates showing the peak for nitric oxide (using DAF-FM probe) and superoxide ion (using MitoSOX Red probe).
