# Supplementary figures and images for: Carnosine Prevents Aβ-Induced Oxidative Stress and Inflammation in Microglial Cells: A Key Role of TGF-β1
Source: Cells. 2019 Jan 17;8(1):64. doi: 10.3390/cells8010064 (PMC6356400; doi:10.3390/cells8010064)

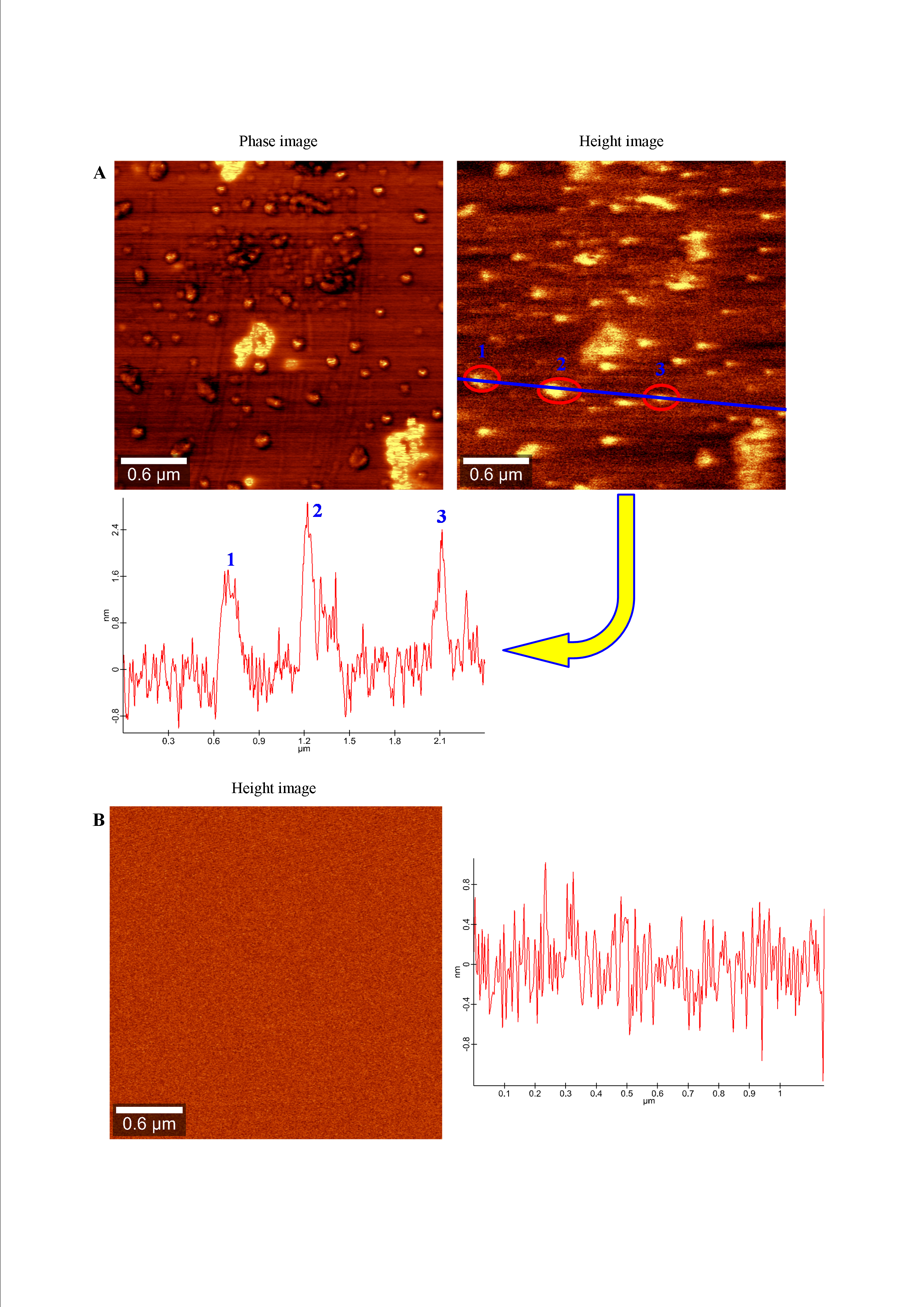

Supplement: Supplementary file 1 [file cells-08-00064-s001.zip › Supplementary Figure S1.tif]

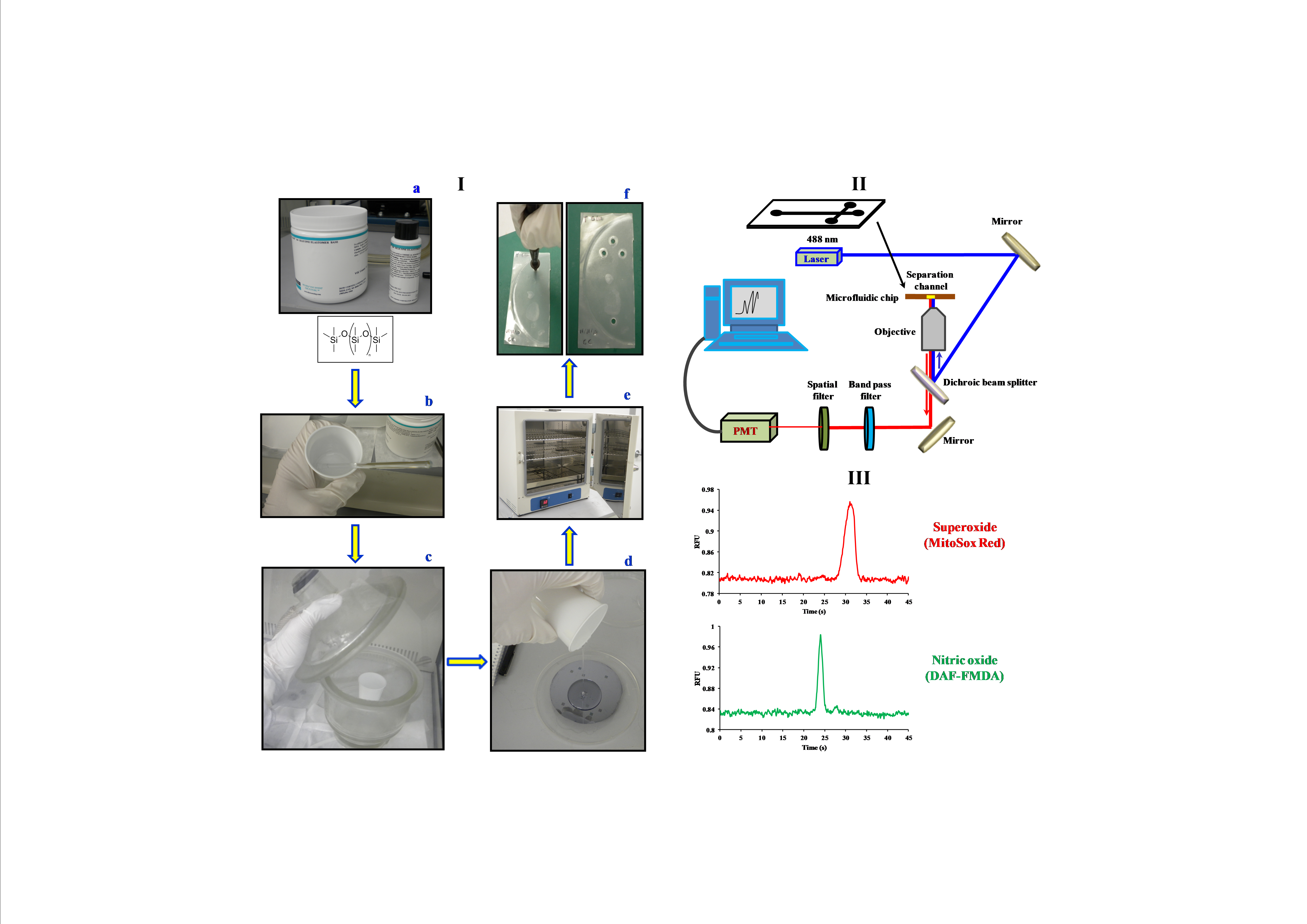

Supplement: Supplementary file 1 [file cells-08-00064-s001.zip › Supplementary Figure S2.tif]
